# Role of cuproptosis-related gene in lung adenocarcinoma

**DOI:** 10.3389/fonc.2022.1080985

**Published:** 2022-12-21

**Authors:** Yuan Liu, Wei Lin, Ying Yang, JingJing Shao, Hongyu Zhao, Gaoren Wang, Aiguo Shen

**Affiliations:** ^1^ Cancer Research Center Nantong, Affiliated Tumor Hospital of Nantong University, Nantong, China; ^2^ Department of Pediatrics, the First Affiliated Hospital, Fujian Medical University, Fuzhou, China; ^3^ Department of Radiotherapy, Affiliated Hospital of Nantong University, Nantong, Jiangsu, China

**Keywords:** cuproptosis, lung adenocarcinoma, immune infiltration, prognostic signature, immune microenvironment

## Abstract

**Backgrounds:**

Lung adenocarcinoma (LUAD) is the most common subtype of lung cancer, which is the leading cause of cancer death. Dysregulation of cell proliferation and death plays a crucial role in the development of LUAD. As of recently, the role of a new form of cell death, cuproptosis, and it has attracted more and more attention. As of yet, it is not clear whether cuproptosis is involved in the progression of LUAD.

**Methods:**

An integrated set of bioinformatics tools was utilized to analyze the expression and prognostic significance of cuproptosis-related genes. Meanwhile, a robust risk signature was developed using machine learning based on prognostic cuproptosis-related genes and explored the value of prognostic cuproptosis-related signature for clinical applications, functional enrichment and immune landscape. Lastly, the dysregulation of the cuproptosis-related genes in LUAD was validated by *in vitro* experiment.

**Results:**

In this study, first, cuproptosis-related genes were found to be differentially expressed in LUAD patients of public databases, and nine of them had prognostic value. Next, a cuproptosis-related model with five features (DLTA, MTF1, GLS, PDHB and PDHA1) was constructed to separate the patients into high- and low-risk groups based on median risk score. Internal validation set and external validation set were used for model validation and evaluation. What’s more, Enrichment analysis of differential genes and the WGCNA identified that cuproptosis-related signatures affected tumor prognosis by influencing tumor immunity. Small molecule compounds were predicted based on differential expressed genes to improve poor prognosis in the high-risk group and a nomogram was constructed to further advance clinical applications. In closing, our data showed that FDX1 affected the prognosis of lung cancer by altering the expression of cuproptosis-related signature.

**Conclusion:**

A new cuproptosis-related signature for survival prediction was constructed and validated by machine learning algorithm and *in vitro* experiments to reflect tumor immune infiltration in LUAD patients. The purpose of this article was to provide a potential diagnostic and therapeutic strategy for LUAD.

## 1 Introduction

As the highest mortality rate and incidence of the second highest rate in the world, lung cancer has a 5-year survival rate of only 26% (1). Lung adenocarcinoma (LUAD), one of the most prevalent subtype in non-small cell lung cancer (NSCLC), accounts for approximately 40% of lung cancers (2). Despite rapid advances in treatment options including chemotherapy, radiotherapy and surgery, the prognosis for LUAD-patients remains unsatisfactory. Over the past few years, immune checkpoint inhibitor (ICI) therapy has emerged as a revolutionary form of cancer treatment that works by targeting immune checkpoints (3). And yet, only a fraction of patients had achieved expected benefit from ICI therapy. To optimize the prognosis and benefit of LUAD pharmacotherapy, reliable biomarkers are required in the era of individualized therapy. Classical clinical models predict the prognosis of LUAD predicted by tumor extension, performance status, TNM staging and pathological staging indicators, but the heterogeneity of LUAD had prevented these models from achieving satisfactory results (4). Therefore, new models need to be constructed for the treatment and prognosis of LUAD.

Proliferation and death of cells are dysregulated in LUAD. Massive cell death is often a precursor to disease progression, followed by an imbalance between cell proliferation and death resulting in tumor growth (5). According to a recent research in Science, an accumulation of intracellular copper ions triggered the aggregation of Fe-S cluster proteins and destabilization of mitochondrial lipidated proteins, leading to a unique type of cell death called cuproptosis (6). On the one hand, elevated copper levels in LUAD patients could promote tumor angiogenesis, progression and metastasis (7). On the other hand, mitochondria could influence cancer drug resistance, leading to poorer chemotherapy outcomes in LUAD patients (8).

The current prevailing doctrine is that treatment failure in lung adenocarcinoma is the result of resistance to apoptosis (9). Although several studies have validated the ability of cuproptosis-related genes to affect the prognosis of lung adenocarcinoma patients by bioinformatics techniques, the mechanism has not been fully elucidated (10–13). Therefore, it might be important to explore the role of cuproptosis-related genes in LUAD.

In the current work, five prognostic cuproptosis-related genes (PCRGs) were identified in LUAD. A signature based on PCRGs was constructed and validated for clinical applications and related mechanisms by machine learning algorithm and *in vitro* experiments. Our findings were expected to provide a valuable diagnostic and therapeutic strategies for LUAD patients.

## 2 Materials and methods

### 2.1 Data collection and sample pre-processing for LUAD patients

RNA sequencing, survival data, and clinical phenotypes were collected from The Cancer Genome Atlas (TCGA) database in University of California Santa Cruz (UCSC) Xena platform (http://www.genome.ucsc.edu/). After data cleaning and normalization, a total of 442 tumor tissues and 49 paracancerous tissues were enrolled. Meanwhile, the normal tissues from Genotype-Tissue Expression (GTEx) (https://www.gtexportal.org) were obtained. In addition, a total of 332 patients with survival time and mRNA expression matrix were download from GSE31210 (246) and GSE30219 (86) in Gene Expression Omnibus database (GEO, https://www.ncbi.nlm.nih.gov/geo/), and 301 patients with survival time clinical data were collected from Affiliated Tumor Hospital of Nantong University.

### 2.2 Consensus clustering

Unsupervised clustering was performed using The “ConsensusClusterPlus” R package. A consensus clustering approach (Euclidean distance) was run by K-means clustering algorithm for 1000 times with a resampling rate of 80%. An empirical cumulative distribution function plot was used to Identified the optimal number of clusters.

### 2.3 Construction of cuproptosis-related signature

First, univariate Cox regression identified PCRGs in the TCGA-LUAD cohort. Subsequently, least absolute shrinkage and selection operator (LASSO) algorithm were performed on the PCRGs in TCGA-LUAD cohort. Finally, the cuproptosis-related signature was constructed by the stepwise Cox regression algorithm. Risk score = $\sum_{i=1}^{n}=$ Coef (Gene) × Expr (Gene). Coef is the coefficient, Expr is the Fragments Per Kilobase of exon Model per million mapped fragments (FPKM) of each gene. LUAD patients with risk-score above the median were categorized as the high-risk subgroup, and the rest were included in the low-risk subgroup. For model, the time-dependent receiver operating characteristic (ROC) was calculated across validation datasets. Survival analysis was used “survival”, “survminer” and “timeROC” packages, and the nomogram was used “rms” package. The ROC curve, the Harrell’s concordance index (C-index), calibration curve and detrended correspondence analysis (DCA) were performed to evaluate the performance of the nomogram.

### 2.4 Identification of differentially expressed genes

The threshold value for screening differential genes was set to |log2FC| > 1 and adjusted *P* < 0.05. The threshold for stepwise-Cox analysis was set to *P* < 0.1 to screen the final pcrg. Paired samples of cancer tissue and paraneoplastic tissue from TCGA were used to test spatial differences in gene expression in the same individual. Human Protein Atlas database was used for the purpose of examining the expression of cuproptosis-related signature at the protein level (https://www.proteinatlas.org/).

### 2.5 Weighted correlation network analysis

The co-expression network of TCGA-LUAD was generated using the weighted gene weighted gene co-expression network analysis (WGCNA) package. A suitable soft threshold β is calculated based on the criteria for scale-free networks. In the following step, the weighted adjacency matrix was converted into a topological overlap matrix (TOM), and the corresponding dissimilarity (1-TOM) was calculated. Module identification was conducted using the dynamic tree cutting approach. The modules most relevant to the clinical phenotype of the risk score were selected for subsequent analysis.

### 2.6 Gene network and enrichment analysis of PCRGs

The gene network analysis was performed using GENEMANIA to analyze potential interactions between these genes (http://genemania.org/). In order to explore the potential mechanisms and pathways between the riskscore subgroups and two clusters, the Gene ontology (GO), Kyotoencyclopedia of genes and genomes (KEGG) functional enrichment analysis, and gene set enrichment analysis (GSEA), gene set variation analysis (GSVA) were conducted among DEGs between the riskscore subgroups and clusters using the R packages “clusterProfiler”, “enrichplot”, “limma”, “ggplot2”, and “org.Hs.eg.db”.

### 2.7 Analysis of immune infiltration

“CIBERSORT” algorithm was used to assess the infiltration of 22 immune cells. Four other algorithms such as xcell, MCP-counter, GSVA, and ESTIMATE, were used to verify the robustness of CIBERSOR algorithm. The immune checkpoints were based on published research (14). The expression of HLA gene were used to assess the capabilities of antigen-presentation response.

### 2.8 Therapeutic response and drug prediction

In order to estimate the likelihood of drug interactions, the DEGs was identified from the high- and low-risk subgroups. L1000FWD (https://maayanlab.cloud/L1000FWD/) database was used to detect the signaling pathways affected by small molecule drugs. PubChem database was used for visualizing the structure of drugs (https://pubchem.ncbi.nlm.nih.gov).

### 2.9 Clinical specimens

Written informed consent was obtained from all patients involved in this study. The ethics committee of the Affiliated Tumor Hospital of Nantong University approved this study.

#### 2.9.1 Quantitative real time PCR

The LUAD and paired para-cancerous samples were collected from 4 patients who underwent lobectomy between August 2022 and October 2022 at Affiliated Tumor Hospital of Nantong University. RNA were extracted and reverse transcription as previously described (15). After reverse transcription, quantitative real time PCR (qRT-PCR) was performed. The primer sequences were shown in **Table 1**.

**Table 1:** Primers used qRT-PCR detection.

GENE	PRIMER	SEQUENCE (5’-3’)
PDHA	Forward	tac agg atg atg cag act gta c
	Reverse	caa gtg aca gaa acc acg aat a
PDHB	Forward	gac act ccc ata tca gag atg g
	Reverse	ctt ggc agc tga gtt tat aac c
GLS	Forward	cac tca aat cag gat tgc g
	Reverse	cca gac tgc ttt tta gca ctt t
MTF1	Forward	gtg cca act ctg tcc taa cta a
	Reverse	cta ctg gta ctg cag tgg taa a
DLAT	Forward	ggg tta ttg cac agc gat taa a
	Reverse	gaa gaa ttt gct tcg gga act t
GAPDH	Forward	act ccc att ctt cca cct ttg
	Reverse	ccc tgt tgc tgt agc cat att

#### 2.9.2 Tissue microarray construction and immunohistochemistry

Tumor and paracancer tissue microarray from Affiliated Tumor Hospital of Nantong University was used for the validation cohort. Primary Anti-FDX1 antibody (1:200; 12592-1-AP, proteintech, China) were applied for the immunohistochemical (IHC) staining. A microscopy system (Nikon, Japan) was used to scan immunohistochemistry sections. We measured the density of positive staining. The H-score was evaluated by two independent pathologists without the knowledge of clinicopathological information.

### 2.10 Molecular docking

The 3D structures of mitoxantrone were downloaded from PubChem database (https://pubchem.ncbi.nlm.nih.gov/). The 3D structure of FDX1 was downloaded from the PDB protein database (https://www.rcsb.org/). Further, the protein was dehydrated and ligand extracted. Then, we conduct molecular simulation docking for the mitoxantrone and FDX1.

### 2.11 Statistical analysis

A complete set of data processing, statistical analysis, and plotting was carried out in R 4.0.3 software. In the case of normally distributed data, the unpaired Student t-test was used, while in the case of non-normally distributed data, the Wilcoxon test was used. An assessment of the correlation between two continuous variables was conducted using Pearson’s correlation coefficients. Univariate Cox regression and multivariate Cox regression were used to detect the effect of factors on prognosis of LUAD,. *P* < 0.05 was regarded as statistically significant.

## 3 Results

The overall design of this study is displayed in **Figure S1**.

### 3.1 Expression of cuproptosis-related genes in carcinoma and adjacent tissues

**Figure 1:**
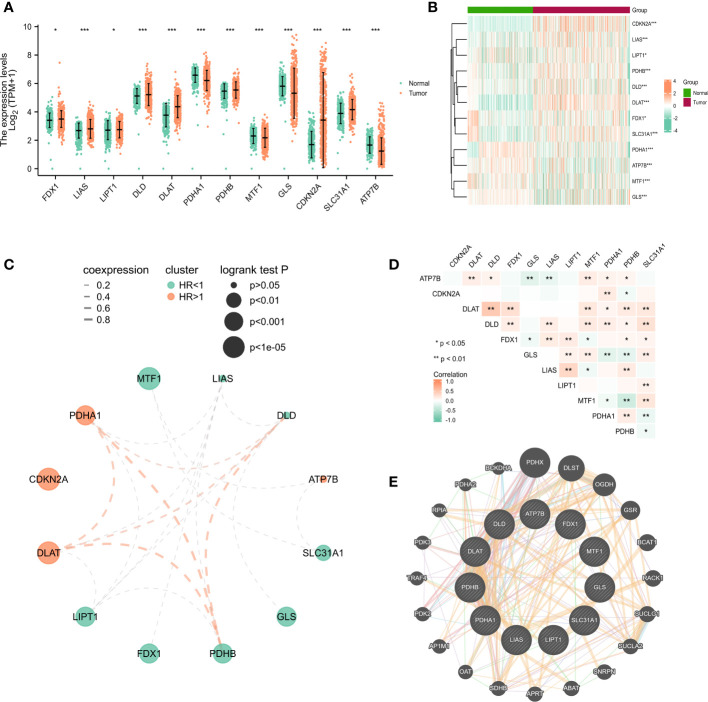
The expression of cuproptosis-related genes in LUAD patients and their prognostic value. Box plots **(A)** and heatmap **(B)** of cuproptosis-related genes in LUAD when compared to normal tissues (with green and red signifying low and high expression levels, respectively). **(C)** Spearman correlation and prognostic values of cuproptosis-related genes in LUAD patients. Red represents HR>1 whereas green represents HR<1. The larger the circle, the smaller the log-rank *p*. **(D)** Correlation analysis between cuproptosis-related genes in LUAD patients. **(E)** Protein-protein interactions among the cuproptosis-associated genes. The bigger the circle is, the most important gene it might be. ^*^ *P*<0.05, ^**^ *P*<0.01, ^***^ *P*<0.001.

**Figure 2:**
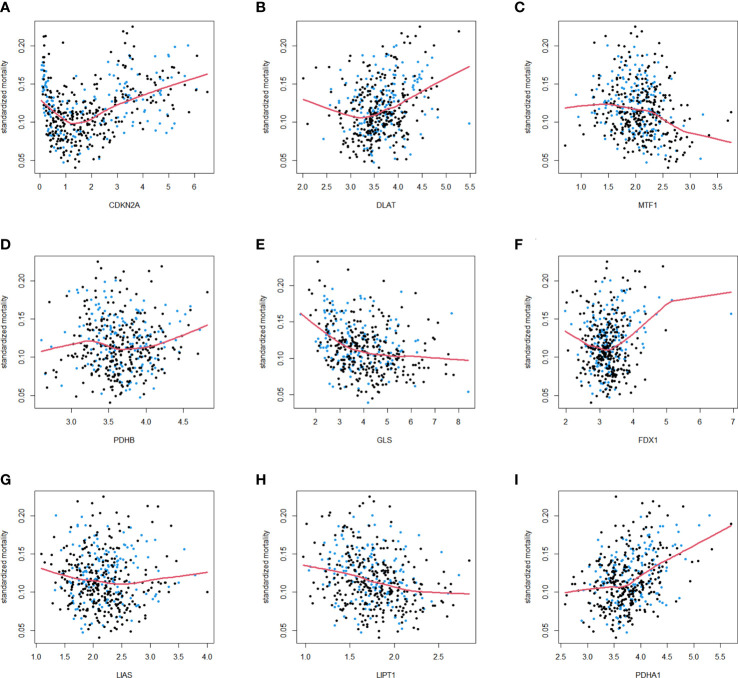
Standardized mortality of 9 cuproptosis-related genes in the TCGA cohort. The standardized mortality of CDKN2A **(A)**, DLAT **(B)**, MTF1 **(C)**, PDHB **(D)**, GLS **(E)**, FDX1 **(F)**, LIAS **(G)**, LIPT1 **(H)**, PDHA1 **(I)** in TCGA cohort. Blue dots correspond to events, and black dots indicate censor.

As described in the literature, 12 genes including FDX1, LIAS, LIPT1, DLD,DLAT, PDHA1, PDHB, MTF1, GLS, CDKN2A, SLC31A1, and ATP7B, were confirmed to be associated with cuproptosis (6). These 12 genes were confirmed in LUAD by comparing their expression patterns in normal and tumor tissues from the TCGA and GTEx databases. It turned out that the expression of these 12 genes was significantly different in both tumor tissues and normal tissues (**Figures 1A, B**). Kaplan-Meier plotter shows 9 genes associated with lung adenocarcinoma prognosis and screened for subsequent analysis (**Figure S2**). Co-expression analysis combined with prognosis value indicated that the expression of 4 cuproptosis-related genes (PDHB, DLAT, PDHB, DLD) was positively co-expressed with each other, and 9 genes (FDX1, LIPT1, DLAT, PDHA1, PDHB, MTF1, GLS, CDKN2A and SLC31A1) had prognostic value in LUAD (**Figure 1C**). Thereafter, we found a significant correlation between the expression of 12 cuproptosis-related genes in the TCGA cohort of LUAD patients (**Figure 1D**). The protein-protein interactions network among the cuproptosis-associated genes were displayed in **Figure 1E**. In parallel, we mapped the association of prognosis-related genes in the TCGA cohort with the standardized mortality rates (**Figure 2**).

### 3.2 Consensus clustering analysis of prognostic cuproptosis-related genes

**Figure 3:**
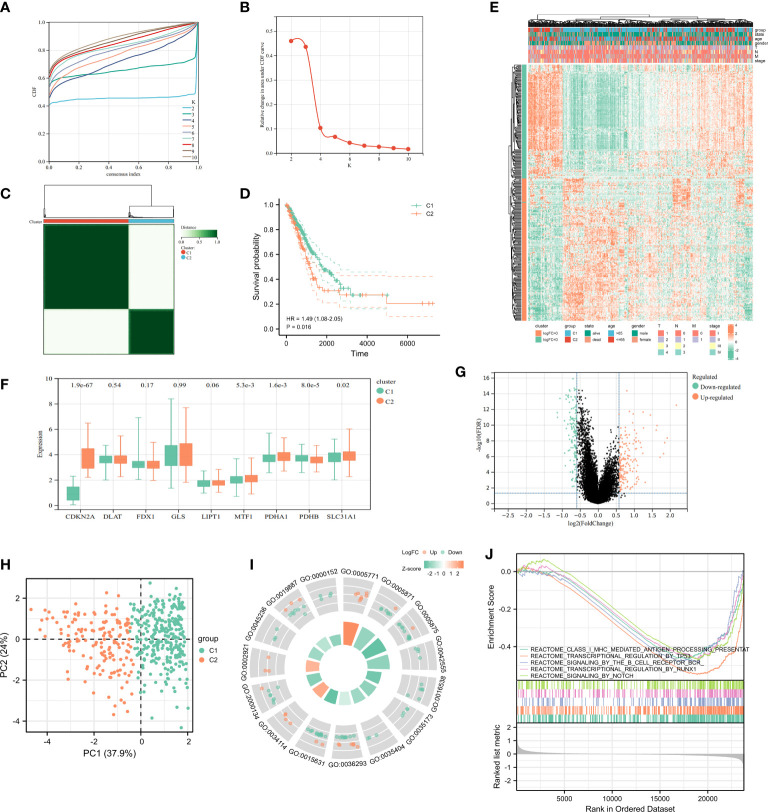
Characteristics of Cuproptosis-related Cluster in TCGA-LUAD cohort. **(A)** Delta area curve of consensus clustering indicated the relative change in area under the cumulative distribution function (CDF) curve from k = 2 to 10. **(B)** The intragroup correlations were the highest and the inter-group correlations were low when k = 2. **(C)** Cluster diagram for consensus clustering analysis (k = 2) of cuproptosis-related genes in 442 LUAD samples in TCGA. **(D)** Kaplan-Meier curve showed survival probability of cluster1 and cluster2. **(E)** The heatmap showed the relationship between clinical features and the expression of cuproptosis-related genes in two clusters. **(F)** The expression of 9 cuproptosis-related genes in two clusters. **(G)** The volcano plot showed the different expression of genes between the two clusters. **(H)** PCA analysis for the two clusters. The most significant GO enrichment **(I)** and multiple pathways by GSEA enrichment analysis **(J)** in two clusters.

In a consensus clustering analysis of 442 tumor specimens from the TCGA cohort, 9 cuproptosis-associated genes were compared to the expression of LUAD subtypes to investigate the relationship between the two. Based on the cumulative distribution function curves of the consensus score matrix (CDF) (**Figure 3A**) and the proportion of ambiguous clustering statistics (PAC) (**Figures 3B, C**), k=2 was the optimal number. When a pair of samples has a high consensus score, they are more inclined to be clustered into the same cluster in successive iterations. Consequently, the 442 patients were divided into cluster 1 (n = 288) and cluster 2 (n = 154). Subsequently, a significant difference in OS was observed between patients in two clusters (**Figure 3D**). The expression of CDKN2A, MTF1, PDHA1, PDHB, SLC31A1 was significantly different between clusters 1 and 2 (**Figure 3F**). The distribution patterns from PCA analysis showed that samples could completely be distinguished into cluster1 and cluster 2 (**Figure 3H**). The heatmap showed the relationship between the different expression of genes and clinical characters (**Figure 3E**), and the volcano plot showed the logFC and FDR value among these genes (**Figure 3G**). There were 169 upregulated genes as well as 137 downregulated genes between the two clusters. The GSEA enrichment analysis found different expressed genes of the two cluster were enriched in some pathways, including signaling of antigen processing presentation mediated by class I MHC, transcription regulation of TP53, signaling of B cell receptor BCR and so on (**Figure 3J**). In addition, the GO enrichment analysis of these genes showed that they were also enriched in certain molecular processes, including histone-serine phosphorylation, response to decreased oxygen levels, multivesicular body, CXCR chemokine receptor binding, etc. (**Figure 3I**).

### 3.3 Establishment, evaluation and validation of prognostic signature based on PCRG in TCGA-LUAD

**Figure 4:**
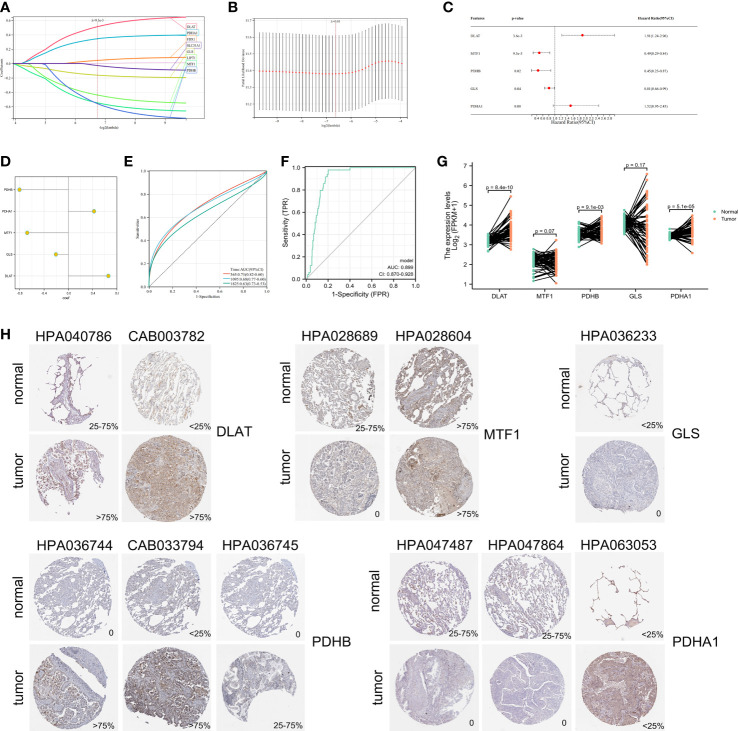
Identification of cuproptosis-related signature *via* LASSO-stepwise algorithms. **(A, B)** LASSO analysis with minimal lambda value. **(C)** Five genes were screen out by stepwise Cox algorithm. **(D)** Coefficients of 5 cuproptosis-related genes finally obtained in stepwise Cox regression. **(E)** The time-dependent ROC curve for Lasso-stepwise signature. **(F)** The ROC curve for LASSO-stepwise signature. **(G)** MRNA expression values in paired samples in TCGA. **(H)** Protein expressions of 5 differentially expressed cuproptosis-related signature in the tumor and normal tissues from the Human Protein Atlas platform.

A prognostic model was constructed based on cuproptosis-related genes. In Cox univariate analysis, nine genes associated with cuproptosis were found to be significantly associated with overall survival (OS) in LUAD patients. Eight cuproptosis-related genes were tested and screened out by LASSO analysis (**Figures 4A, B**). Five genes were extracted using stepwise regression to construct the model (**Figure 4C**). In order to develop a risk-score model, the following algorithm was used: Risk score = (0.659854097) * DLAT + (-0.204720564) * GLS + (-0.67817864) * MTF1 + (0.423421209) * PDHA1 + (-0.805217147) * PDHB. The coefficients of the five PCRGs were displayed in **Figure 4D**, and TCGA-LUAD patients was divided into high-riskscore (n = 221) and low-riskscore (n =221) subgroups according to the median to facilitate the next step of study. As a measure of the signature’s specificity and sensitivity, areas under curve (AUC) values were calculated for 1-, 3- and 5 years. They were 0.71, 0.68 and 0.63 in TCGA-training set (**Figure 4E**). In addition, we found that the signature had not only a prognostic value but also a good diagnostic value (**Figure 4F**, AUC=0.899). We detected the MRNA expression of cuproptosis-related signature in TCGA paired samples and found the expression of DLAT, PDHB, PDHA1 were significantly increased in cancer tissues when compared with cancer-adjacent tissues, while the expression of GLS and MTF1 trended downward, the decrease was not significant between paracancerous tissues and cancer tissues (**Figure 4G**). In the Human Protein Atlas database, the expressions of GLS, MTF1 and PDHA1 in tumor tissues were lower compared with those in normal tissues, while the expressions of DLAT and PDHB were significantly higher in tumor tissues compared with those in normal tissues (**Figure 4H**). Notably, all antibodies in the database were used for each gene to avoid false positives.

**Figure 5:**
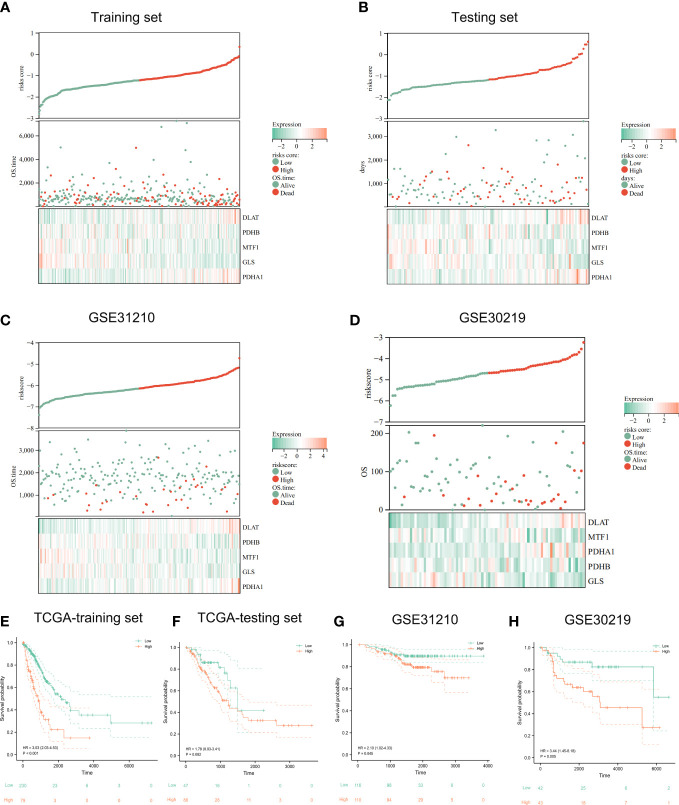
Evaluation and validation of prognostic signature. The risk-score, survival time, survival status and gene expression of the training set **(A)**, testing set **(B)**, and external set GSE31210 **(C)** and GSE30219 **(D)**. Kaplan-Meier analysis demonstrated the prognostic significance of the risk model in TCGA training set **(E)**, testing set **(F)** and GSE31210 **(G)**, GSE30219 cohort **(H)**.

The risk survival status charts and Kaplan–Meier (K-M) survival analysis showed that survival time of LUAD patients in the low-risk group was longer than that of LUAD patients in the high-risk group, not only in TCGA training set (**Figures 5A, E**), but also in TCGA internal testing set (**Figures 5B, F**) and external verification set (GSE31210 and GSE30219) (**Figures 5C, D, G, H**).

### 3.4 Distribution of prognostic riskscores and prognosis stratified by clinical characteristics

**Figure 6:**
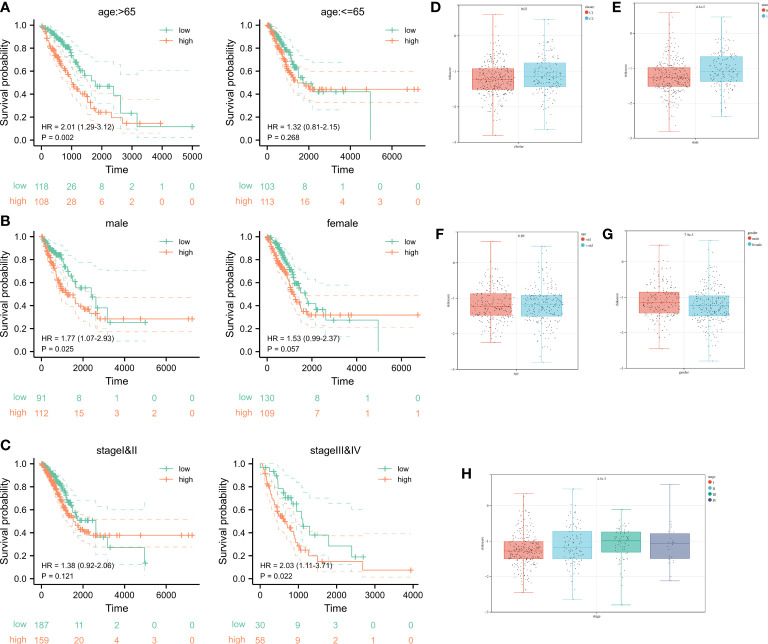
Survival analysis after stratification of clinical characteristics and distribution of clinical characteristics after risk stratification. **(A-C)** Kaplan–Meier curves and the log-rank test showed that the overall survival of the high-risk set was worse than that of the low-risk set in age and gender subgroups of patients. **(D-H)** The distribution of riskscore in clusters, status, age, gender, as well as pathological stage.

According to K-M curves, the OS of the high-risk set was significantly worse than that of the low-risk group in age > 65 subgroups. In clinical subgroups of age ≤ 65, prognosis was not significant after riskscore stratification (**Figure 6A**). After stratifying for the characteristic variable of gender, it was found that the OS of the high-risk set was worse than that of the low-risk set in gender subgroups of patients (**Figure 6B**). In addition, stratification of pathological staging yielded resulted that the OS of the high-set was significantly worse than that of the low-risk set in stages III and IV subgroup (**Figure 6C**). Even though there were no significant difference in the OS of low and high risk sets in age ≤ 65, female, stage I and II subgroups, its overall OS trend was consistent with that seen before stratification. We speculated that this might be due to the lower sample size after stratifying samples and resulting in less statistical power.

Ultimately, even in different clinical subgroups, LUAD patients in the high-risk group had a lower survival probability than those in the low-risk group. In the subgroups after K-means clustering, it showed a significant difference in the distribution of riskscore, which proved the consistency of K-means clustering resulted with cuproptosis-related signature results (**Figures 5E-H**). Combining the analysis results of the K-means algorithm, we found that the riskscore was higher in Cluster 2 compared to the Cluster 1 (**Figure 6D**). The distribution of riskscores differed significantly between survival outcomes, suggesting that those who occurring ending events were more likely to be from a high-risk group (**Figure 6E**). There was no significant difference in the distribution of the riskscore between the two subgroups (aged > 65 years or ≤ 65 years), implying that age was not a factor affecting the distribution of the riskscore (**Figure 6F**). However, gender affected the distribution of the riskscore, with female patients having lower riskscore values compared to male patients (**Figure 6G**). It was noteworthy that the riskscores were higher as the tumor development and pathological stage changed (**Figure 6H**). The above results suggest that after stratification of clinical features, our riskscore distribution influenced prognostic outcomes, and our signature has high specificity and sensitivity in predicting the prognosis of LUAD patients.

### 3.5 Identification of cuproptosis-related modules derived from riskscore patterns

**Figure 7:**
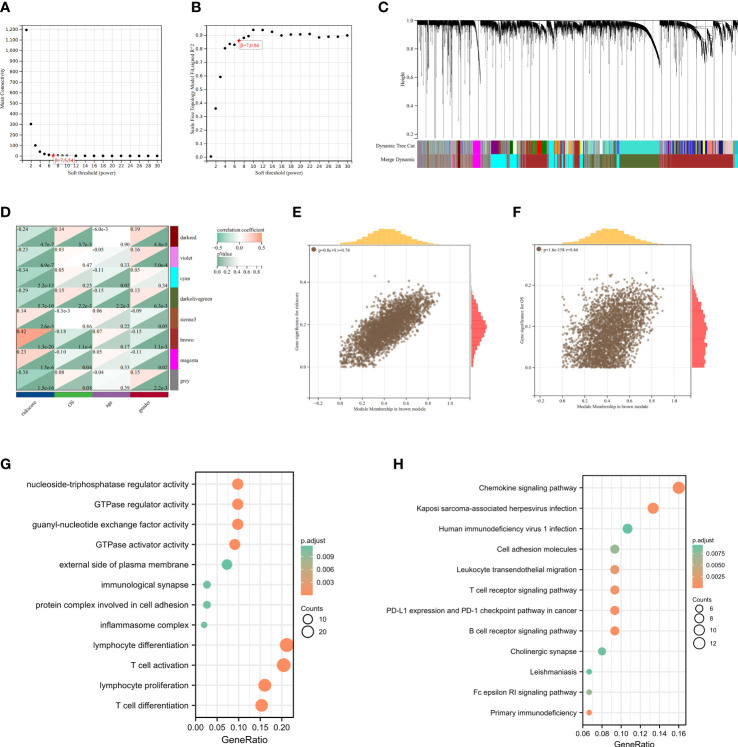
Identification of cuproptosis-related signature *via* WGCNA. **(A)** The correlation between soft threshold and scale free topology model fit signed R2. **(B)** The correlation between soft threshold and mean connectivity. **(C)** Clustering of module feature vectors. **(D)** The correlations between modules and clinical traits were calculated. **(E)** The high correlation between GS and MM in the brown module in riskscore subgroups. **(F)** Genes in the brown module were associated with survival status in TCGA. **(G)** GO enrichment analysis after screening out Hub gene from brown module. **(H)** KEGG enrichnment analysis after screening out Hub gene from brown module.

Setting the soft threshold β to 7 (unsigned, R = 0.86) in the WGCNA could provide a suitable power value for the co-expression network (**Figures 7A, B**). After identifying 8 different modules, the correlation between different color modules and clinical features was calculated separately (**Figures 7C, D**). Brown module and riskscore subgroups exhibited the highest correlation of module-trait relationships. For the brown module, the R value between GS and MM was reached 0.74 in riskscore (**Figure 7E**) and 0.44 in status (**Figure 7F**), which suggested that the cuproptosis-related module was well constructed and the module was significantly associated with prognosis. To identify Hub cuproptosis-related module derived from cuproptosis-related patterns within the brown module, 167 genes with GS > 0.7 and MM > 0.2 were considered hub cuproptosis-related genes. The GO analysis of these hub genes was enriched into the lymphocyte differentiation, T cell activation, lymphocyte proliferation, protein complex involved in cell adhesion, inflammasome complex, immunological synapse, guanyl−nucleotide exchange factor activity, GTPase regulator activity and so on (**Figure 7G**). KEGG analysis enriched these genes into Chemokine signaling pathway, B cell receptor signaling pathway, PD−L1 expression and PD−1 checkpoint pathway in cancer and so on (**Figure 7H**). This suggested that our modules closely related to the cuproptosis-related signature and mainly affected the immunomodulation of patients with LUAD.

### 3.6 Immune infiltration analysis for cuproptosis-signature

**Figure 8:**
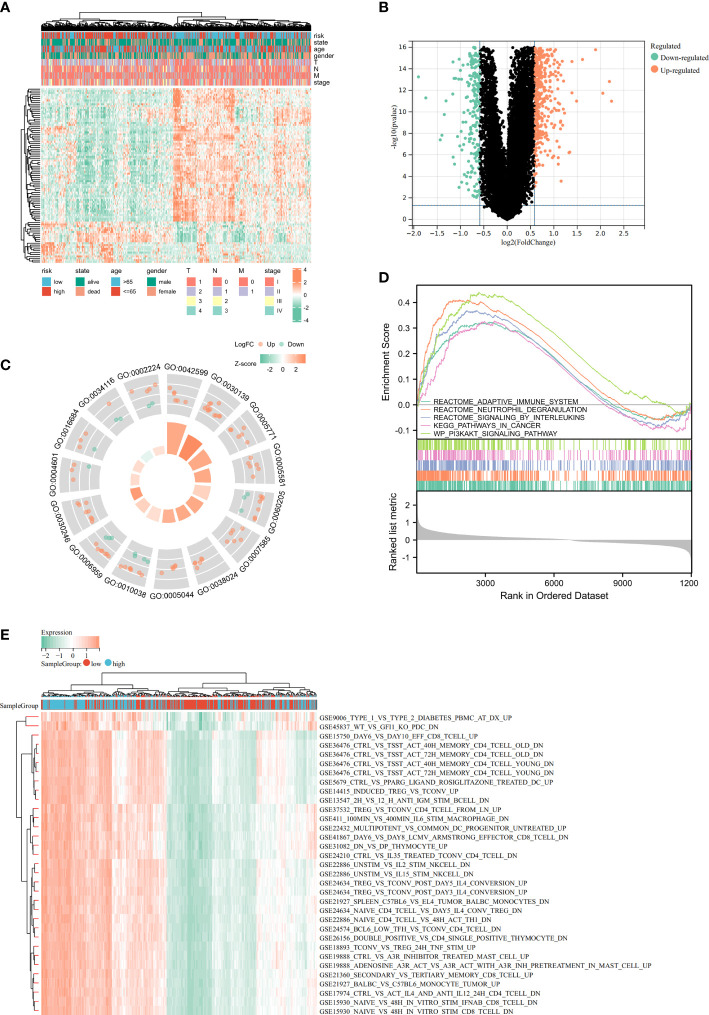
Identification of differentially expressed genes (DEGs) and potential signaling pathways in different isoforms. The heatmap **(A)** and volcano plot **(B)** of the differential gene expression between high and low expressed cuproptosis-related signature in LUAD. GO enrichment **(C)** and GSEA **(D)** analysis of the differential expressed genes. **(E)** GSVA enrichment analysis of the differential genes.

The heatmap showed the relationship between the riskscore subgroups and clinical characters (**Figure 8A**). The volcano plot showed the logFC and FDR value among these different expressed genes (**Figure 8B**).

**Figure 9:**
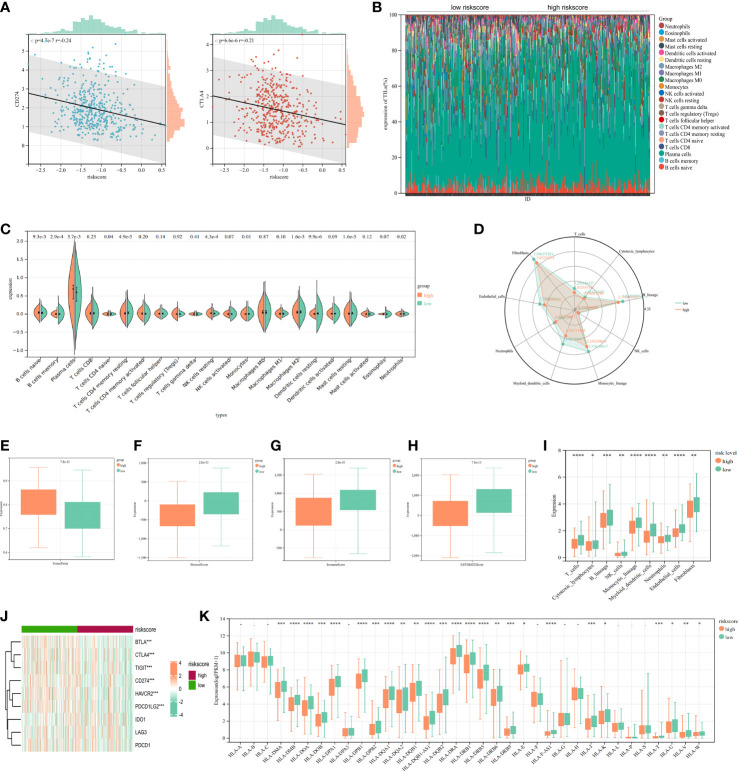
Immune infiltration analysis of signature. **(A)** The correlation analysis of PD-L1/CTL4 expression and riskscore distribution in LUAD. **(B)** The percentage abundance of tumor-infiltrating immune cells showed the immune infiltration analysis between high risk-score and low risk-score in LUAD patients. **(C)** The infiltrating levels of immune cells in high risk-score and low risk-score groups in LUAD patients. **(D)** Xcell algorithms detected immune cell expression between the high-risk and low-risk subtypes. **(E-H)** Comparison of ESTIMATE, stromal, and immune scores between the cluster 1 and cluster 2. **(I)** MCP-counter algorithm calculated the immune infiltrating cell score for each subgroups. **(J)** Comparison of immune checkpoints between high-risk and low-risk subgroups. **(K)** The HLA genes between high-risk and low-risk subtypes. ^*^ *P*<0.05, ^**^ *P*<0.01, ^***^ *P*<0.001, ^****^ *P*<0.0001.

As part of this study, we performed enrichment analyses on GO (**Figure 8C**), GSEA (**Figure 8D**), and GSVA (**Figure 8E**) to elucidate the underlying functions and pathways associated with our prognostic features. The results of GO, GSEA, GSVA, and WGCNA all suggested that genes associated with our prognostic features were associated with immune infiltration. Meanwhile, Risk-scores were negatively correlated with CD274 (PD-L1) and CTLA4 expression (**Figure 9A**). We evaluated the differences in infiltration of 22 immune cell types in the riskscore subgroup through the Cibersort database (**Figures 9B, C**). The results showed that B cells naive, Plasma cells, etc. were significantly higher while T cells CD4 memory resting, Macrophages M2, mast cells resting were significantly lower in the high-risk group when compared to the low-risk group. To demonstrate that the functions of the two subgroups were not biased by the analytical algorithm, Xcell (**Figure 9D**), ESTIMATE (**Figures 9E-H**), and MCP-counter (**Figure 9I**) algorithms were used to verify the stability and robustness of the Cibersort result. We further found significant differences in common immune checkpoints between the two subgroups as well (**Figure 9J**). HLA genes are also closely related to tumor immunity (16). Additionally, we examined whether HLA-related genes were expressed differently in risk subgroups. High-risk individuals tended to have lower HLA gene expression than low-risk individuals (**Figure 9K**). Together, these results revealed a higher tumor purity with lower immune cell infiltration in high-risk subgroup, which may affect immunotherapy outcomes in LUAD patients.

### 3.7 Clinical applications for cuproptosis-signature

**Figure 10:**
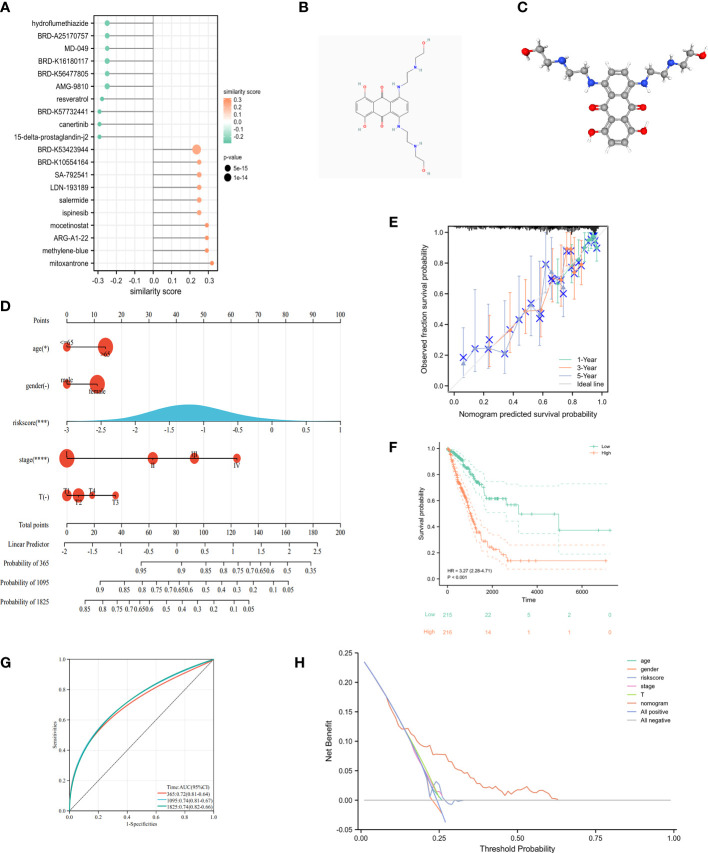
Clinical application of cuproptosis-related signature. **(A)** The potential drug for LUAD treatment. **(B)** The 2D structure of mitoxantrone. **(C)** The 3D structure of mitoxantrone. **(D)** The nomogram of the riskscore and clinical parameters (age, gender, T and pathological stage) of TCGA. **(E)** The calibration curves displayed the accuracy of the nomogram in the 1-, 3-, and 5-years. **(F)** Kaplan-Meier curve in multiple Cox regression analysis. **(G)** The time-ROC curve in multiple Cox regression analysis. **(H)** DCA curves to assess the ability of age, gender, risk score, T stage, and their combination to predict overall survival of LUAD patients in TCGA-LUAD cohort. ^*^ *P*<0.05, ^***^ *P*<0.001, ^****^ *P*<0.0001.

Identification of 20 important small molecules through the L1000FWD database as drugs to improve poor prognosis in high-risk groups (**Figure 10A**). Mitoxantrone was predicted to be the most promising drug. We further visualized the 2D (**Figure 10B**) and 3D (**Figure 10C**) structure of Mitoxantrone. A nomogram was created by integrating clinical information and genetic features from TCGA and performing multivariate Cox regression models (**Figure 10D**). Calibration plots were applied in OS outcomes, which demonstrated favorable concordance between predicted and observed OS at 1-, 3- and 5-year survival (**Figure 10E**). In terms of prediction, the C-index was 0.70 (0.67-0.73), which reflects relatively good performance. Also, LUAD-patients with high score had worse survival than those with low score (**Figure 10F**). The AUC values of 1-, 3- and 5-year in nomogram were 0.72, 0.74 and 0.74, respectively (**Figure 10G**). Additionally, we evaluated the nomogram model in TCGA-LUAD using decision curve analysis (DCA) (**Figure 10H**). Altogether, risk score was an independent and good prognostic indicator, and LUAD patients could benefit more after combining with pathological stage, T-stage and risk scores.

### 3.8 Validation of *in vitro* experiments and molecular docking

**Figure 11:**
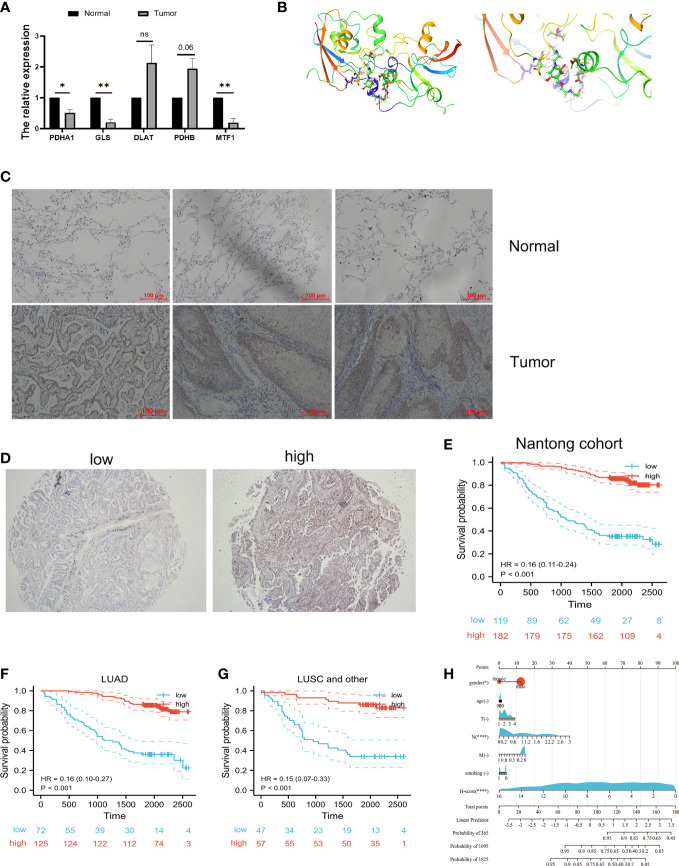
Validation of vitro experiment and molecular docking. **(A)** Validation of mRNA expression in prognostic cuproptosis-related signature by qRT-PCR. ^*^ *P*<0.05, ^**^ *P*<0.01. **(B)** The molecular docking between FDX1 and mitoxantrone. **(C)** The IHC results showed that FDX1 protein is highly expressed in tumor tissues when compared with normal tissues. **(D)** Representative immunohistochemical microarray of FDX1. **(E)** Kaplan–Meier curves showed that the overall survival of the low-risk set was worse than that of the high-risk set in Nantong cohort. **(F, G)** Kaplan–Meier curves showed that the overall survival of the low-risk set was worse than that of the high-risk set in LUAD **(F)** and lung squamous cell (LUSC) subgroups **(G)** of patients. **(H)** The nomogram of the H-score and clinical parameters (age, gender, smoking and T, N, M stage) of Nantong cohort. ns, not significant.

To confirm the role of cuproptosis-signature in LUAD, we further verified their differential expression in normal and tumor samples by vitro experiments. qRT-PCR was performed in paired samples of cancer and paracancer to detect mRNA expression levels of the prognostic cuproptosis-signature. We found that the expression of PDHA1, GLS, DLAT, PDHB and MTF1 were differed between cancer and paracancer (**Figure 11A**). We have deciphered that the small molecule compound mitoxantrone could treat the risk of death due to immune tolerance induced by cuproptosis-related signature. FDX1 as an enzyme catalyzing cuproptosis-related signature (17), we further used molecular docking to verify the direct interaction of FDX1 with the small molecule compound mitoxantrone (**Figure 11B**).

Next, tissue microarray of 301 lung cancer patients from the Nantong Cancer Hospital were used in a cohort study. FDX1 expression was identified by immunohistochemical staining in 301 lung cancer samples. We observed that FDX1 was located in the cytoplasm of tumor cells (**Figure 11C**). Immunohistochemical score (H-score) for immunostaining of tumor tissues also differed in each sample (**Figure 11D**). 119 (39.5%) patients were classified into FDX1 low expression subgroup while the FDX1 high expression subgroup had 182 (60.5%) patients. In our cohort, the patients with high FDX1 expression had significantly better outcomes than those with low FDX1 expression (**Figure 11E**). Stratification by pathological type revealed that the high expression group of FDX1 had a better prognosis than the low expression group in both LUAD and other lung cancer patients (**Figures 11F, G**). Most importantly, we constructed Nomogram and found that H-score was one of the most significant independent predictors of OS (**Figure 11H**). The Harrell’ s c-index for the nomogram model to predict the overall survival was 0.81. In a word, our study showed that FDX1, a key enzyme for cuproptosis, affected the prognosis of lung cancer patients by influencing the expression of GLS, PDHA1, PDHB, DLAT, and MTF1.

## 4 Discussion

Globally, lung cancer is the leading cause of death among cancer patients. Approximately 40% of all the diagnosed cases were LUADs (2). Immune escape and drug resistance are the major drivers of cancer death and begin when cancer cells invade surrounding tissues. With the recent advent of molecularly targeted therapies and immunotherapies, survival in LUAD has been highly improved. However, drug resistance and recurrence remain the main causes of tumor progression in patients with LUAD and are influenced by factors inherent to immune cells, cancer cells, or both (18). The immune escape and resisting tumor cell death are hallmarks of cancer as well as the basis for acquiring resistance following immunotherapy (19, 20). New discoveries of programmed cell death patterns and the elucidation of related molecular mechanisms continually update our knowledge of cell death in tumors. Recently, a new form of cell death with copper-dependence, called cuproptosis, was first proposed by Tsvetkov et al (17). There have been several studies showing an association between copper metabolism and tumorigenesis, as well as a higher copper demand by cancer cells than by normal cells, and that dysregulation of copper ions is significantly associated with drug resistance (21). Liao et al. considered that copper metabolism might be responsible for the development of colorectal cancer with an immune response (7). Currently, the specific mechanism of cuproptosis-related genes in LUAD remains unclear.

In our study, we first found that 8 cuproptosis-related genes were upregulated, while 4 genes were downregulated in LUAD when compared to normal tissues. Then, the K-means algorithm divided the cohort into two clusters, and the set of genes associated with cuproptosis in LUAD was mainly enriched in MHC-I mediated antigen processing expression, transcriptional regulation of TP53, and signaling of the B cell receptor BCR in cluster 2, whose OS was obviously poorer than that in cluster 1. These above results suggested that the prognostic differences between cuproptosis-related clusters were associated to immune response. There were five PCRGs derived from univariate regression, LASSO, and stepwise regression. Then after, five PCRGs were used to construct a novel prognostic risk signature, which stratified LUAD patients into high- and low-risk subgroups. Differential cuproptosis-related mRNA expression was verified in TCGA paired samples, and protein expression of cuproptosis-related signature was further verified in Human Protein Atlas platform. The prognostic signature integrated 5 PCRGs, including DLAT, MTF1, PDHB, GLS and PDHA1. Among them, DLAT and PDHA1 were associated with poor prognosis of LUAD while MTF1, PDHB and GLS were associated with good prognosis of LUAD.

DLAT (dihydrolipoamide S-acetyltransferase) is expressed in mitochondria which involved in cell glycolysis. A number of malignancies, including lung cancer (22), gastric cancer (23) and colon cancer (24), have been associated with high expression of DLAT in various tumors. These studies suggested a potential role of DLAT in abnormal cuproptosis metabolism in LUAD. Similarly, copper-induced cell death was mediated by protein lipoylation. PDHA1 (pyruvate dehydrogenase E1 subunit alpha 1) as a hub gene in Fatty acid metabolism pathway, played an important role in cuproptosis. Chen et al. found that high expression of PDHA1 in NSCLC promoted tumor progression *in vivo* and *in vitro* (25). Notably, PDHA1 was highly expressed in LUAD by Human Protein Atlas platform analysis, which was opposite of mRNA expression in TCGA, indicating that PDHA1 might not regulated in mRNA level in LUAD. Interestingly, MTF1, PDHB and GLS were reported to be highly expressed in other tumor types, while in our study, we found that these genes were strongly associated with good prognosis. MTF1 (metal regulatory transcription factor 1) responds sensitively to both metal excess and deficiency, protects cells from oxidative and hypoxic stresses (26), is dysregulated in cancer and pain disease (27). As a consequence of the mitochondrial respiratory and functional impairment in LUAD, cells lacking MTF1 are more sensitive to oxidative stress. PDHB (pyruvate dehydrogenase E1 subunit beta) encodes a subunit of the pyruvate dehydrogenase complex, which converts pyruvate to acetyl-CoA in the mitochondrion (28). Zhu et al. found that PDHB was associated with tumor growth and metastasis and glycolysis (29). GLS (glutaminase) is oncogenic and can influence the metabolic reprogramming of cancer through its functional selective genome and epigenome (30). A recent study showed that in pancreatic ductal adenocarcinoma (PDAC), activated GLS increased glutamine catabolism and production of nicotinamide adenine dinucleotide phosphate (NADPH) and glutathione, which prevented from being oxidative and promoting tumor cell survival and tumor growth in mice (31). Taken together, all of the five crucial genes were involved in tumorigenesis and progression by regulating pathways associated with tumor metabolism. Additionally, our data further elucidated the important role of these 5 PCRGs in LUAD, and the PCRGs for LUAD patients were highly sensitive and specific.

Immunotherapy has been a powerful clinical strategy for treating cancer (32). PD-1/PD-L1 checkpoint blockade immunotherapy has joined chemotherapy as a standard treatment for lung cancer (33). Unfortunately, the metabolic reprogramming of tumors poses a considerable challenge for cells to perform their immune functions as well as to cancer immunotherapy (34). In particular, cells containing high lipid acylated proteins are more sensitive to copper-induced cell death. Thus, in tumors characterized by high lipid metabolism, the induction of copper death in cells may be able to resolve drug resistance caused by immune escape. As expected, our results of WGCNA algorithm, GO and KEGG analysis were significantly associated with immune response related pathways, including T cell receptor signaling pathway, PD-L1 expression and PD-1 checkpoint pathway in cancer, B cell receptor signaling pathway. Similarly, Limma package further validated that the immune pathways were enriched by cuproptosis-associated genes. Notably, these pathways, such as the adaptive immune system, signaling by GPCR and PI3K/AKT/mTOR signaling have been widely confirmed to be involved in LUAD (35). Taken together, the application of two machine learning algorithms revealed that there was an inextricable connection between copper-dependent cell death and tumor immune responses in LUAD (36).

A major finding of previous study found tumor purity was negatively correlated with immune response and might be a proxy for the level of immune response in the tumor microenvironment (37). Consistent with the finding, in our study, the tumor purity was higher and the immune infiltration was lower in high-risk set when compared with the low-risk one. The abundances of B cells, plasma cells, resting memory T cells CD4, resting NK cells, monocytes, M2 macrophages, resting DCs, resting mast cells, and neutrophils were substantially different between high and low riskscore group. Ample evidence has shown that the infiltration of B cells, especially naive B cells, memory B cells, and plasma cells is associated with a good prognosis of LUAD (38). In contrast, we found the high-risk subgroup had a poor prognosis while high plasma cell expression. It is difficult to explain the discrepancy between these data and our results. Considering the loss of an HLA allele may be a mechanism of immune escape (39), we can only speculate that the decreased expression of HLA genes as well as immune checkpoint genes in the high-riskscore group is likely to be associated with dysregulation of immune cells. More data is needed in the future to support this conjecture.

Afterward, to verify the general applicability of the riskscore subgroups, validation was performed on the internal validation set and the external validation set GSE31210. The signature exhibited good predictive performance for both the internal and external validation sets. ROC curves and Kaplan-Meier curves showed that PCRGs were good predictors of prognosis in LUAD patients. It is worth noting that after stratified analysis of clinical characteristics, the signature still produced significant prognostic differences in the LUAD patients. In other words, the cuproptosis-related signature had good predictive performance in OS and may serve as an independent prognostic indicator for LUAD. Nomogram was constructed to further advance clinical applications and the accuracy of the maps was verified with calibration plots. Small molecule drugs have been now commonly used in the treatment of cancer and widely used in clinical practice (40). Our study identified 20 small molecule drugs thar were most significant for LUAD treatment. The top 2 of them were mitoxantrone and 15-delta-prostaglandin-j2. Mitoxantrone has been found to treat breast cancer through blocking cellular autophagy (41). Whether Mitoxantrone can inhibit tumor development by activating autophagy or copper death in LUAD needs to be further investigated. In addition, 15-delta-prostaglandin-j2 was a natural ligand for PPARγ. Activation of PPARγ is well known to be beneficial in the treatment of breast and colon cancers (42). PPARγ plays a pivotal role in lipid metabolism (43) and protein lipoylation is necessary for copper death. Thus, the role of PPARγ in LUAD deserves further elucidated.

Functionally, FDX1 catalyzes proteolipidylation of a battery of substrates, as the most prominent marker of cuproptosis. Previous bioinformatic analysis of cuproptosis-related genes is mainly at the plane of RNA transcriptional regulation (44–46), ignoring the fact that the function of proteases depends mainly on protein expression and activity. As far as we know, our study was the first investigation illustrating the correlation between FDX1 protein expression and unfavorable prognosis of lung cancer patients by tissue microarray. FDX1 as the bridge between unfavorable prognosis of lung cancer patients and cuproptosis.

Mechanically, Mo. et al. found that lncRNA MIR31HG/miR-193a-3p/TNFRSF21 axis may indirectly regulates the occurrence of cuproptosis in lung adenocarcinoma. Wang et al. considered lncUCA1/miR-1-3p/DLD axis leading to the occurrence of cuproptosis (47). These studies involve in pre-transcriptional regulation and lack direct favorable evidence for the role of cuproptosis phenomenon in the prognostic outcome of lung adenocarcinoma patients. Chen et al. discovered the alterations in mRNAs such as BARX1, ENTP2 were found to be associated with the influence of elesclomol (48). These experiments on these cells lines only indirectly demonstrate the occurrence of possible cuproptosis-related mRNA alterations at the cellular level. We constructed a cuproptosis-related signature by bioinformatic analysis, in which five genes serve as direct substrates of FDX1 and their alterations are the most significant markers of cuproptosis. FDX1 affects the prognosis of lung cancer by altering the expression of cuproptosis-related signature (GLS, PDHA1, PDHB, MTF1, and DLAT), which more strongly confirms that the prognosis of lung cancer patients is closely related to the occurrence of cuproptosis.

Several limitations are worth mentioning. Sincerely, the hypothesis needs to be further validated by more research. To begin with, the study only included cohorts from TCGA, GEO and Nantong chort. It is not possible to fully assess the quality of the data, to further evaluate the prognostic cuproptosis-signature in the future, a prospective, multicenter study is required. Lastly, to further clarify the mechanism and function of the cuproptosis-signature in tumorigenesis and LUAD progression, *in vivo* experiment might be conducted.

In conclusion, the purpose of this research was to develop a prognostic cuproptosis-related signature, which could be used to predict survival of LUAD patients, reflecting the tumor immune infiltration. What’s more, it may be a key to improve the prognosis for LUAD patients through immunotherapy. It is expected that this research will provide new insights into how to diagnose and treat LUAD patients with precision.

## Data availability statement

The datasets presented in this study can be found in online repositories. The names of the repository/repositories and accession number(s) can be found in the article/**Supplementary Material**.

## Ethics statement

The studies involving human participants were reviewed and approved by the ethics committee of the Affiliated Tumor Hospital of Nantong University. The patients/participants provided their written informed consent to participate in this study.

## Author contributions

A formal analysis was conducted and the initial draft was written by YL. The article was written, reviewed, and edited by WL, YY, JS, GW, and HZ contributed to the conception of this research. AS supervised all experiments. All authors contributed to the article and approved the submitted version.
