# Supplementary figures and images for: Role of cuproptosis-related gene in lung adenocarcinoma
Source: Front Oncol. 2022 Dec 21;12:1080985. doi: 10.3389/fonc.2022.1080985 (PMC9811388; doi:10.3389/fonc.2022.1080985)

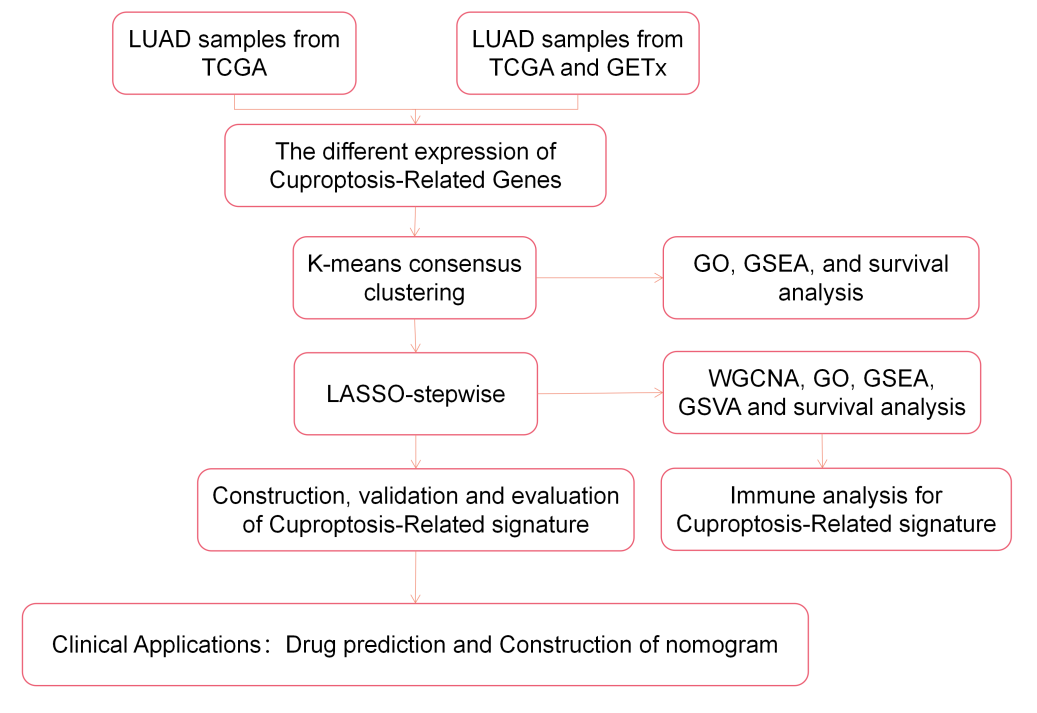

Supplement: Supplementary file 1 [file Image_1.tif]

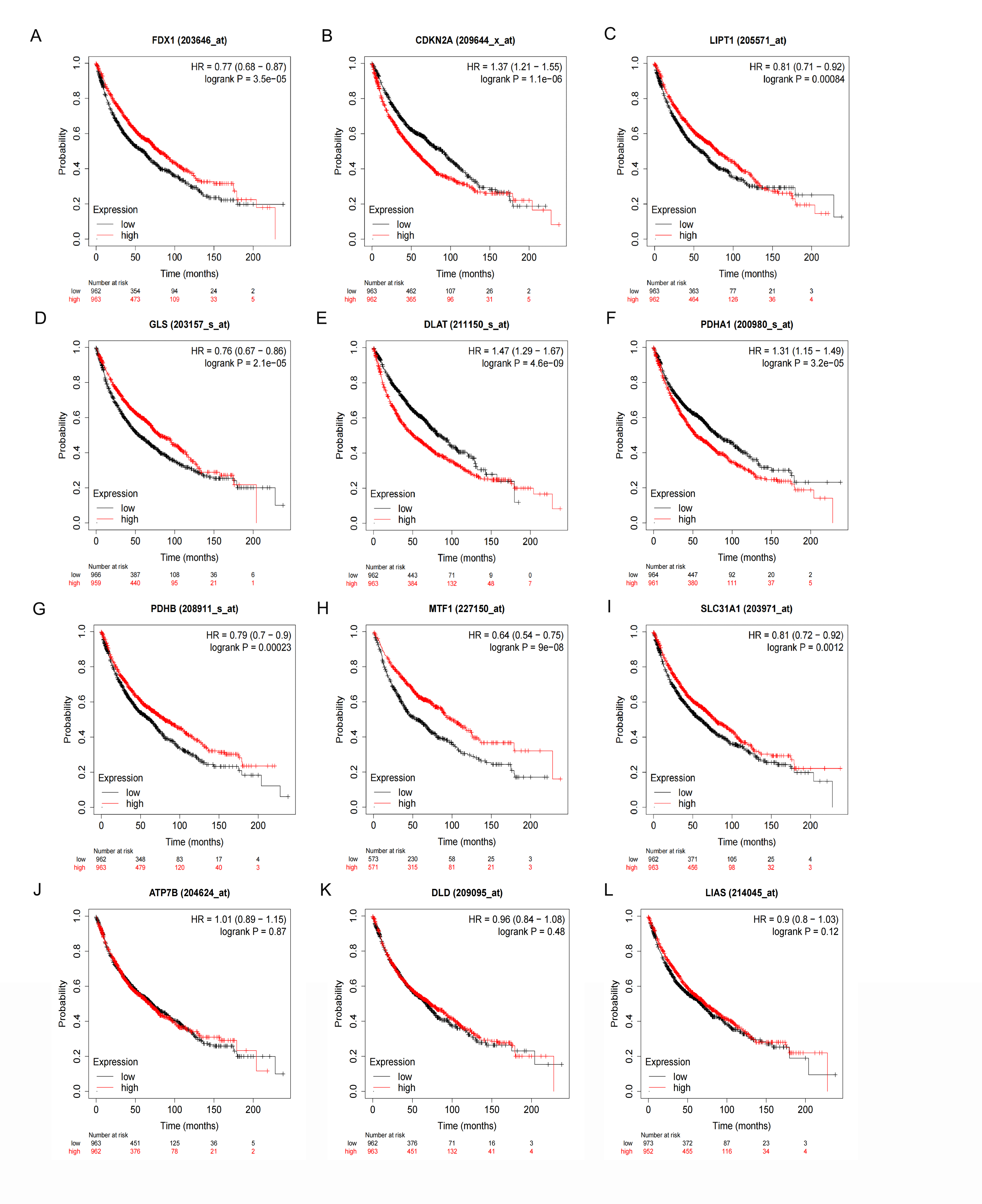

Supplement: Supplementary file 2 [file Image_2.tif]
